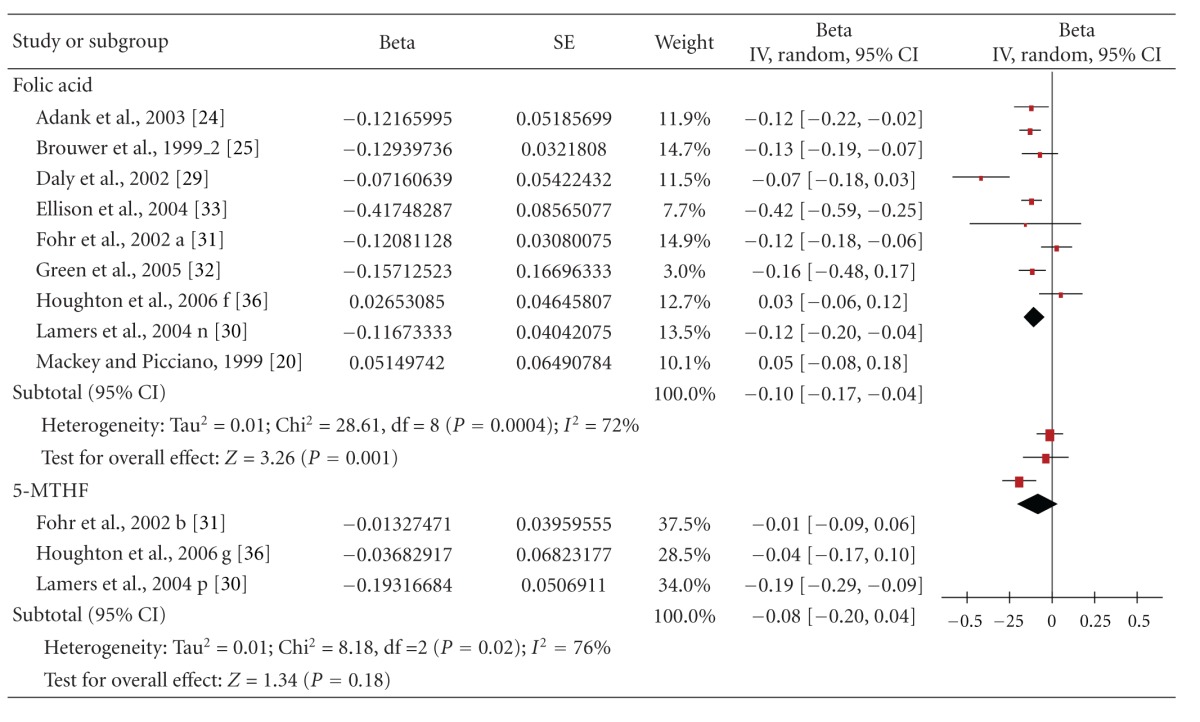# Folate Intake and Markers of Folate Status in Women of Reproductive Age, Pregnant and Lactating Women: A Meta-Analysis

**DOI:** 10.1155/2012/470656

**Published:** 2012-09-13

**Authors:** Cristiana Berti, Katalin Fekete, Carla Dullemeijer, Monica Trovato, Olga W. Souverein, Adriënne Cavelaars, Rosalie Dhonukshe-Rutten, Maddalena Massari, Tamás Decsi, Pieter van't Veer, Irene Cetin

**Affiliations:** ^1^Unit of Obstetrics and Gynecology, Department of Clinical Sciences Hospital ‘L. Sacco' and Center for Fetal Research Giorgio Pardi, University of Milan, 20157 Milan, Italy; ^2^University of Pécs, H-7623 Pécs, Hungary; ^3^Division of Human Nutrition, Wageningen University and Research Centre, P.O. Box 8129, 6700 EV Wageningen, The Netherlands

## Abstract

*Background*. Pregnant and breastfeeding women are at risk for folate deficiency. Folate supplementation has been shown to be associated with enhanced markers of folate status. However, dose-response analyses for adult women are still lacking. *Objective*. To assess the dose-response relationship between total folate intake (folic acid *plus* dietary folate) and markers of folate status (plasma/serum folate, red blood cell folate, and plasma homocysteine); to evaluate potential differences between women in childbearing age, pregnant and lactating women. *Methods*. Electronic literature searches were carried out on three databases until February 2010. The overall pooled regression coefficient (**β**) and SE(**β**) were calculated using meta-analysis on a double-log scale. *Results*. The majority of data was based on nonpregnant, nonlactating women in childbearingage. The pooled estimate of the relationship between folate intake and serum/plasma folate was 0.56 (95% CI = 0.40–0.72, *P* < 0.00001); that is, the doubling of folate intake increases the folate level in serum/plasma by 47%. For red blood cell folate, the pooled-effect estimate was 0.30 (95% CI = 0.22–0.38, *P* < 0.00001), that is, +23% for doubling intake. For plasma-homocysteine it was –0.10 (95% = –0.17 to –0.04, *P* = 0.001), that is, –7% for doubling the intake. Associations tended to be weaker in pregnant and lactating women. *Conclusion*. Significant relationships between folate intake and serum/plasma folate, red blood cell folate, and plasma homocysteine were quantified. This dose-response methodology may be applied for setting requirements for women in childbearing age, as well as for pregnant and lactating women.

## 1. Introduction

Folate is involved in one-carbon transfer reactions, which are fundamental for DNA and RNA synthesis, amino acid metabolism, and formate oxidation [1]. Plasma or serum folate, red blood cell (RBC) folate, and homocysteine (tHcy) are used as biomarkers of folate status and reflect reliably changes in folate intakes [2, 3]. Serum folate reflects primarily recent intake, whereas erythrocyte folate levels reflect time-integrated intake and are considered to be a measure of long-term folate status [4]. Plasma homocysteine is an indicator of low or deficient folate status, and therefore considered as an indicator of folate adequacy [5] on the basis that normal homocysteine metabolism requires an adequate supply of folate [6].

Folate intake and status play a crucial role during pregnancy [7]. Pregnant and breastfeeding women are at risk for folate deficiency due to an increased need for folate. Previously, a longitudinal study showed low folate status among participants, especially in late pregnancy and during lactation [3]. Moreover, a meta-analysis including twenty-one controlled trials on folate supplementation in pregnant women showed that compared to placebo or no supplementation, folate supplementation was associated with strongly increased serum folate levels and red cell folate levels [8]. Regarding breastfeeding, a review of the literature indicates that apparently healthy women, who do not receive folic acid supplementation, can already become folate depleted in the early postpartum [9]. 

Deriving a dose-response association may help to address the above issues and identify major modifiers of the intake-status relationship, useful to address questions on the “optimal” level of a folate biomarker and to recommending intake of folic acid/folate for mother's and newborn's health. Actually, the adequate folate intake during pregnancy has not been clearly stated yet. Six hundred *μ*g/day DFEs (Dietary Folate Equivalents) are considered sufficient to maintain adequate folate status in pregnant women [10–12]. Nordic Nutrition Recommendations [13] recommend 500 *μ*g/day DFE while the dietary reference intakes of total folate are 400 *μ*g/day in Italy [14] and 300 *μ*g/day in UK [15]. Internationally, women are advised to use folic acid supplements during the periconceptional period on the basis of the well-recognized link between maternal folate status and neural tube defects (NTDs) [16]. Some debate also exists regarding the proper daily dosage of folic acid/folate for preventing NTDs [17]. The current recommendation to supplement 400 *μ*g folic acid daily starting at least 4 weeks before conception which seems to be not enough to achieve optimal-red-cell-folate levels within 4 weeks, because this requires at least 8–12 weeks of daily intake [18]. In contrast, with 800 *μ*g folic acid, the health criterion is reached within an average of 4 weeks after the start of supplementation [19]. Regarding lactating women, for example, Mackey and Picciano [20] found that folate status in lactating women was preserved with 1 mg/day of supplemental folate for three months.

However, the strength of the dose-response relationship between folate intake and folate biomarkers has not been established yet, except for the relation with risk of NTDs in women aged 20–65 years [21]. Consequently, we used meta-analysis to model folate status as a function of supplemental folate intake. We conducted a meta-analysis of data from randomised controlled studies (RCTs) that examined the effect of folate exposure on folate status in women in childbearing age, pregnant and lactating women, in order to quantitatively assess folate dose-response relationships. 

## 2. Methods

This research is part of a project within the European Micronutrient Recommendations Aligned (EURRECA) Network of Excellence that aims to identify micronutrient requirements for optimal health in European populations [22]. The review here reported was part of a wider review process to identify studies assessing the effect of folate intake on both different markers of folate status as well as health outcomes. 

## 3. Electronic Searches

The methodology here used is based on a standard methodology developed for EURRECA reviews [23]. A protocol was provided outlining specific linkages among the populations of interest, exposures, modifying factors, biological role of folate, and outcomes of interest, in order to define study eligibility criteria prior to starting the literature search and in order to interpret relevant studies once they are identified. The general search strategy included terms for randomised controlled trials in humans AND (intake or status) AND (folate or folic acid or vitamin B_9_). Electronic searches were carried out over all years until February 2010. Both indexing and text terms were used and each search strategy was further adapted for the individual databases searched (Ovid EMBASE, Ovid Medline, and Cochrane Central). The reference lists of collected articles and of published reviews were also checked for relevant studies to be included into the screening and data extraction process.

## 4. Data Collection

The results of the searches were combined, and papers were screened on the basis of title and abstract; references clearly not meeting the review criteria were excluded. This task was divided between two independent reviewers (K. Fekete and C. Berti), with a minimum of 10% overlapping in order to harmonise the process. Once potentially relevant literature was identified, full-text articles were retrieved and reviewed for inclusion on the basis of the predetermined inclusion/exclusion criteria (Table 1). Only papers meeting all criteria were included and extracted onto an Access database by a single reviewer (C. Berti). Corresponding authors of papers were contacted to obtain values or data when data in the original articles were not clear or presented as graph. Information pertaining to bibliographic details, and study characteristics, as well as nutrient supplement information such as intake/dose, source of micronutrient, chemical form, mode of delivery, duration of delivery, chemical analysis, measures of prior nutritional status, level of the nutrient in the background diet, method used to estimate intake, and analytical methods used to assess nutrient status were collated.

## 5. Assessment of Internal Validity 

In order to assess the risk of bias of the studies, the following indicators of internal validity specific to the RCT methodology were collected during data extraction: (1) method of sequence generation and allocation, (2) blinding, (3) potential funding bias, (4) number of participants at start, (5) dropouts and dropout reasons, (6) dose check, (7) dietary intake data reported, (8) outcome comparability and reproducibility, and (9) similarity of most and least exposed groups at baseline. Based on these indicators, two reviewers decided on the overall risk of bias. Disagreements were resolved by discussion. The criteria for judging these indicators were adapted from the Cochrane Handbook [38].

## 6. Data Analysis

The effect of total folate intake (folic acid plus dietary folate), expressed as *μ*g/d DFEs [39], was investigated through meta-analysis of the intervention group compared with the control group for all included studies that assessed the specified biomarkers of folate status. Based on the assumption that the bioavailability of folic acid added to food is greater than that of natural food folate by a factor of 1.7, the amounts of folic acid from supplements/fortified foods were transformed into amounts of folate by multiplying × 1.7 [6, 39]. In the studies evaluating the effects of supplements or fortified foods, when dietary intake was not provided, the mean dietary folate intake of 247 *μ*g/day from other comparable studies was used in the calculation. If required, concentration data expressed as nmol/l were converted to the SI units conventional *μ*g/L (i.e., ng/mL) by dividing by the conversion factor (i.e., 2.266). 

We calculated an intake-status regression coefficient ($\hat{\beta }$) and the corresponding standard error (SE) for each individual study [40]. The intake-status relationship was assumed to be linear on the ln-ln-scale (natural logarithm of intake versus natural logarithm of status). This assumption is based upon our hypothesis that within the range of observations, the true intake-status relationship for folate can be described by a monotonic concave curve, which slowly grows to positive infinity as intake increases and rapidly goes to negative infinity as intake approaches 0. We calculated the overall pooled *β* and its SE using random effects meta-analysis, which estimates the between-study variance using the method of DerSimonian and Laird [41] and uses this estimate to modify the weights used to calculate the summary estimate. Residual heterogeneity between studies was evaluated using the I^2^ statistic. A priori defined subgroup analyses according to dose, duration of supplementation, and population group were carried out to try to explain the heterogeneity. Meta-analysis was carried out with Review Manager (RevMan) 5.0 (Copenhagen: The Nordic Cochrane Centre, The Cochrane Collaboration, 2008).

## 7. Results

**Figure 1:**
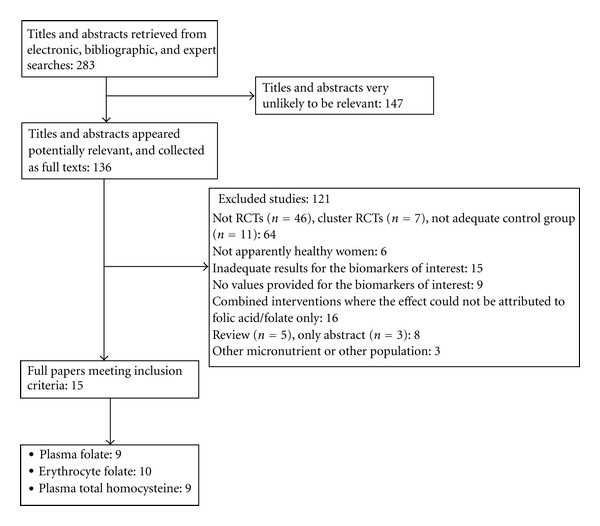
Flow diagram of the articles screened, assessed, and excluded at various stages for this paper.

Figure 1 shows the flow diagram outlining the search results for pregnant and lactating women. Of the overall 4067 hits recovered through the general search, after the removal of duplicates and titles and abstracts highly unlikely to be relevant for the aims of this paper (data not shown), a total of 283 titles and abstracts were potentially available. After inclusion of studies from bibliographic searches, title and abstract were screened, 136 were collected as full-text articles, and 15 articles fulfilled the inclusion criteria. Papers were excluded because they were review articles, or dealt with not interventional studies, or with trial inappropriately designed or with intervention studies including other population groups or nonhealthy women or without the minimal duration or dealt with combined intervention where the effects could not be attributed solely to folic acid. Also, papers with incomplete data not obtainable from the authors were excluded. 

Characteristics of the included studies are summarised in Table 2. Eight studies compared the placebo group with more than one intervention group [18, 24–31, 36]. The majority of interventions assessed the effect of folic acid from supplements (*n* = 14), while only one used fortified foods [32]. Four studies [18, 30, 31, 36] were designed as three-arm trials, comparing placebo to both folic acid and 5-methyltetrahydrofolate or [6S]-5-methyltetrahydrofolate (5-MTHF) or racemic MTHF. We analysed the two types of supplement separately.

Ten studies included women in childbearing age, three were on pregnant women [33–35] and two included lactating women [20, 36]. Placebo was used in the control group in the majority of studies except for two studies [27, 33] in which the control group did not undergo any treatment. Only one study included pregnant adolescents [35]. Most of the trials (i.e., ten) were conducted in Europe, two in New Zealand [24, 32], one was in Brazil [35], one in Canada [36], and one in the USA [20].

The majority of the studies (e.g., 12 out of 15) presented a moderate risk of bias (data not shown). The most common reason for this was an overall lack of information about the method of randomization (i.e., inadequate or unclear sequence generation and/or allocation) and/or unclear source of funding. In contrast, most of the trials reported reasons for dropouts and numbers of dropouts, as well as information on compliance (i.e., methods, number of no compliants or dose check).

## 8. Serum/Plasma Folate

We identified nine studies that reported the effect of specified doses of folic acid up to 1.0 mg/day *plus* dietary folate on serum/plasma folate. Three trials were in pregnant women, four in women in childbearing age, and two studies during the postpartum period. Overall, 632 participants were included in the studies with a duration ranging from 4 to 25 weeks. For further details on the characteristics of included studies, see Table 2.

The forest plot of serum/plasma folate response to folic acid *plus* dietary folate supply is shown in Table 4. The overall pooled estimate was 0.56 (95% CI = 0.40–0.72), and a significant effect of folate on serum/plasma concentrations was demonstrated (*P* < 0.00001). This means that a 2-fold higher folate intake corresponds to a 1.47-fold higher serum/plasma folate, that is, a 47% increase. The test for heterogeneity showed high heterogeneity among the studies. When subgroup analyses were carried out, heterogeneity remained high within these subgroups (Table 3). The results of the meta-analysis derive mainly from the trials on women in childbearing age which represented half of all subjects.

When the effect of 5-MTHF *plus* dietary folate was quantified in three studies in a total of 221 women, we found an overall pooled estimate (95% CI) of =1.18 (0.65, 1.71); *P* < 0.0001 (Table 4). A doubling of 5-MTHF intake will lead to increase serum/plasma folate levels by 2.26-fold, that is, 126%.

## 9. Red Blood Cell Folate

Ten trials contained eligible data regarding the effects of folic acid up to 1.0 mg/day *plus* dietary folate on (RBC) folate. These trials included 724 women. Supplements were taken between 4 and 24 weeks. These trials primarily involved women of childbearing age (seven trials), whereas only one trial included pregnant women and two trials included women during the post partum period. For further details on the characteristics of included studies, see Table 2. As demonstrated in Table 5, pooling response to folic acid/folate supplementation in one meta-analysis yielded an overall pooled Beta (95% CI) of 0.30 [0.22, 0.38]; *P* < 0.00001). This means that doubling the intake of folic acid leads to an 23% increase of RBC folate concentrations. Primary analysis was highly heterogeneous. Stratified analyses did not reduce substantially the level of heterogeneity (Table 3). The results of the meta-analysis are highly influenced by the trials with women of childbearing age, because they represented the majority of subjects (486 out of 724) included in the meta-analysis.

Among the 10 studies, three also administered 5-MTHF *plus* dietary folate in a total of 221 women. When combined in meta-analysis, the overall pooled estimate was 0.49 (0.20, 0.77) (*P* = 0.0008) (Table 5).

## 10. Plasma tHcy 

We identified nine studies that reported the effect of specified doses of folic acid up to 1.0 mg/day *plus* dietary folate on plasma tHcy. The intervention duration ranged from 4 to 24 weeks. These trials were mostly conducted in women in childbearing age (six trials), one was conducting during pregnancy, and two studies were in women during the postpartum period. For further details on the characteristics of included studies, see Table 2. The primary analysis of the trials (Table 6) suggested that daily folic acid/folate was significantly inversely associated with tHcy concentrations (overall pooled Beta; Beta-random effect (95% CI) = −0.10 (−0.17, −0.04); *P* = 0.001, *n* = 585). Consequently, a doubling of folate intake lowers the levels of tHcy by 7%. The heterogeneity was high and therefore subgroup analysis was conducted (Table 3). This showed that heterogeneity was lower in the subgroup of trials in women in childbearing age and breastfeeding, as well as in the subgroup of trials with a duration of 4–12 weeks of supplementation. Again, the majority of subjects in the meta-analysis were women of childbearing age.

Of the studies, three also evaluated the effect of 5-MTHF *plus* dietary folate in a total of 221 women. An inverse, but not statistically significant association, was found between 5-MTHF and plasma tHcy (overall pooled Beta [95% CI] = −0.08 [−0.20, 0.04]) (Table 6).

## 11. Discussion

The amount of nutrients needed to prevent deficiencies, to maintain body stores, and to reduce the risk of chronic diseases represent the basis for establishing micronutrient recommendations. Our meta-analysis was designed to quantify the dose-response relationship between folate intake and biomarkers of folate status. This information is useful to decide what dose of folate or folic acid to recommend for women planning a pregnancy, and subsequent lactation. Previously, only Wald et al. [21] provided a quantitative estimate of the dose-response relation between folic acid intake, in doses of up to 1 mg/day, and risk of NTD assessed. They found that serum folate concentrations increase by 0.94 *μ*g/L (95% CI = 0.77–1.10) for every 0.1 mg/day increase in folic acid intake in women aged 20–35 years. We applied a base-e logarithmic transformation on the folate intake up to 1 mg/day and markers of folate status. The overall Beta represents the difference in the ln-transformed predicted value of serum/plasma folate status per one-unit difference on the ln-transformed scale of folate intake. Therefore, the relative change in intake to the power$\;\hat{\beta \;}$represents the relative change in the biomarker concentration. For example, the overall pooled Beta of 0.56 for women means that a 1.4-fold increase of the mean intake from 250 to 350 *μ*g/day corresponds to a 1.21-fold increase in plasma folate, that is, from an average of 16.0 to 19.3 nmol/L (=1.21 ∗ 16), that is, 3.3 nmol/L per 100 *μ*g/day, which is about 1.5 times stronger than the estimated 2.13 nmol/L per 0.1 mg/day as estimated by Wald et al. [21]. Such a difference may be explained by considering both the characteristics of the studies included and the methodological approach used. Firstly, Wald and colleagues [21] included trials with and without a placebo/control group in their analysis whilst we used only data from randomized controlled trials. Moreover, our review involved more updated references with respect to the papers they evaluated, and only two studies were included in our as well as their meta-analysis. Finally, Wald and colleagues evaluated the effect for given doses of folic acid, not for total folate intakes (i.e., folic acid plus dietary folate). 

The main effect found by pooling data from the selected RCTs showed that folic acid *plus* dietary folate supply exerted a significant effect on all the markers of folate status, which was particularly strong for RBC and plasma/serum folate. Similarly, the meta-analysis by Mohamed [8] showed that routine folate supplementation in pregnancy resulted in a substantial reduction in the incidence of low serum and RBC levels. However, the strength of the dose-response relationships, we observed in pregnant women and in lactating women was weaker than that found within women in childbearing age. During these physiological periods maintaining maternal biomarkers concentration at a given level seems to be more difficult. This may be due to the anabolic needs of pregnancy and the loss via lactation. Based on factorial approaches, folate requirements during pregnancy are 5- to 10-fold higher than in the nonpregnant condition, owing to the enlargement of the uterus, the development of the placenta, the increasing red cell volume of the mother, and the growth of the developing fetus [17]. In breast feeding, milk folate secretion is strictly regulated to keep folate supply at a level that prevents the development of folate inadequacy in infants, but often at the expense of maternal folate stores. Metz and colleagues [42] demonstrated that folic acid was preferentially uptaken by milk compared to serum. Moreover, the same authors found that also in lactating women with severe folate deficiency, oral administration of folic acid appeared to be transferred to breast milk in preference even to the hemapoietic systems [42].

It is worthwhile to note that all the RCTs included in the meta-analysis were studies of supplementation. Supplemental amounts of folate are required to satisfy the increased needs of women planning pregnancy, during pregnancy and breastfeeding. Since natural food folates degrade during food processing, and their bioavailability is low [7], folate intakes from diet are found to be suboptimal from the perspective of achieving an optimal folate status [6]. In contrast, folic acid, that is, the synthetic form of folate, is highly bioavailable and chemically stable, thus it is the most common form of folate used in supplements and fortified foods. Interestingly, by comparing the strength of associations between total folate intake and folate status, we observed that the relationship tended to be stronger for 5-MTHF than for folic acid. Some authors suggested that 5-MTHF was as effective as or more effective than folic acid in preserving folate status [17, 18, 36] given that 5-MTHF is the predominant folate transport and storage form within the body. Use of the naturally occurring folate form 5-MTHF as a possible substitute for folic acid is under consideration because it is unlikely to mask vitamin B-12 deficiency, and does not produce unmetabolized folic acid in the circulation in contrast to folic acid [43]. 

The main strength of this meta-analysis is the selection of data from randomized controlled trials. Ideally, RCTs should provide reliable data about the effect of an intervention. This means that changes in folate indicators are definitely due to folic acid/folate intervention. However, it has to be taken into account that after the Medical Research Council Vitamin Study [16] studies designed to assess the effect of folic acid intake on measures of folate status in the periconceptional period cannot be studied in controlled trials due to ethical reasons. Folic acid supplementation is recognized indispensable around conception in protecting against NTDs because the neural tube closes between 23rd and 27th day of pregnancy. Thereby, the application of our *inclusion criteria* lets us to exclude several studies on this topic because they were not RCTs. Moreover, we found that there is an overall lack of research regarding the role of folate in pregnancy outcomes different from NTDs. Further research is needed to investigate the role of folate supply in the latter two trimesters of pregnancy. An adequate folate intake seems in fact to play an important role in the implantation and development of the placenta, and in improving endothelial function, to suggest that adequate amount of folate might also be beneficial along the entire gestation [7]. Similarly, functional or health outcomes of various folic acid/folate intakes have been rarely explored in lactating women. On the whole, in fact, trials included in this review mostly recruited women of childbearing age, while trials on women during pregnancy and lactation are scarce, suggesting that further research is needed to explore this question. Most of the trials included small size samples. Moreover, our assessment of internal validity showed that most of the studies had a moderate to high risk of bias as assessed by our criteria. We found a great heterogeneity among trials probably due to differences in methodological factors and physiological characteristics of women studied. However, the analysis of the potential influence of folic acid dose, duration of supplementation, or population on the association revealed that these factors did not significantly explain the between-study heterogeneity.

## 12. Conclusion

Statistically significant relationships between total folate intake and serum/plasma folate, red blood cell folate and tHcy were shown. In particular, a doubling of the total folate intake significantly increased the folate concentration level in serum/plasma and RBC by 47% and 23%, respectively, and lowered the levels of plasma tHcy by 7%. This dose-response approach here applied may in future be applied for deriving the intake dose necessary to achieve the optimal level of a folate biomarker for women of childbearing age, as well as for pregnant and lactating women. 

## Figures and Tables

**Table 1:** Inclusion criteria followed to select potentially relevant papers for data extraction.

Population characteristics	Apparently healthy participants at baseline
Study design	Randomised controlled trial
Intervention	Supplements or fortified foods or natural diet intakes versus a placebo or untreated group
Duration	4 weeks
Outcomes	Must report the following relationships: *intake*-*status*
Intake measures	Report of intake from supplements or fortified foods or natural food sources
Status measurements	Red blood cell (RBC) folate Plasma/serum folate Homocysteine (tHcy)
Baseline information	Baseline data must be present for all reported outcomes

**Table 2:** General characteristics of the included studies and effects on the biomarkers of folate intervention in women of childbearing age, pregnant and lactating women, with respect to the control/placebo according to the original paper.

Author, year	Country	Population (*n*. included)	Description of supply	N. in intervention (FA) and in control groups (*P*) at latest time	Study design	Biomarkers reported	*P* value
Adank et al., 2003 [24]	New Zealand	Childbearing age women (239)	Folic acid capsule = 400 *μ*g/day. Folic acid capsule = 2800 *μ*g/week. Placebo capsule	FA (400) = 36 *P* = 44	Double blind. Duration: 12 wks. Dietary assessment	RBC folate (nmol/L) (Microtiter technique, L. Casei). Plasma homocysteine (*μ*mol/L) (fluorescence polarization immunoassay).	<0.001 <0.05

Brouwer et al., 1999_1 [25]	Netherlands	Childbearing age women (144)	Folic acid tablet = 250 *μ*g/day (500 *μ*g folic acid and a placebo tablet every second day). Folic acid tablet = 500 *μ*g/day. Placebo tablet.	FA (250) = 50 FA (500) = 45 *P* = 49	Double blind, 2 containers of indistinguishable tablets: one red marked, one yellow marked. Duration: 4 wks. Dietary assessment	RBC folate (nmol/L) (Imx automated immunoassay system). Plasma folate (Imx automated immunoassay system).	FA (250): ^a^ <0.01 FA (500): ^a^ <0.001 FA (250): ^a^ <0.001 FA (500): ^a^ <0.001

Brouwer et al., 1999_2 [26]	Netherlands	Childbearing age women (144)	Folic acid tablet = 250 *μ*g/day (500 *μ*g folic acid every the 2nd day). Folic acid tablet = 500 *μ*g/day. Placebo tablet.	FA (250) = 50 FA (500) = 45 *P* = 49	Double blind, 2 containers of indistinguishable tablets: one red-marked, one yellow-marked. Duration: 4 wks. Dietary assessment	Plasma homocysteine (*μ*mol/L) (HPLC and fluorimetric detection)	FA (250): ^a^ <0.01 FA (500): ^a^ <0.001

Cuskelly et al., 1996 [27]	UK	Childbearing age women (49)	Folic acid capsule = 400 *μ*g/day. Folic-acid-fortified foods plus 400 *μ*g/day (FA-f). Dietary folate plus 400 *μ*g/day (FA-d). Dietary advice (DA) Control group: no treatment	FA = 9 FA-f = 6 FA-d = 10 DA = 7 *P* = 9	Duration: 12 wks mos. Dietary assessment	RBC folate (*μ*g/L) (microbiological assay)	

Daly et al., 1997 [28]	Ireland	Childbearing age women (110)	Folic acid tablet = 200–400 *μ*g/day. Placebo tablet	FA (100) = 22 FA (200) = 28 FA (400) = 26 *P* = 19	Double blind, identical tablets. Duration: 24 wks	RBC folate (*μ*g/L) (microbiological assay)	

Daly et al., 2002 [29]	Ireland	Childbearing age women (110)	Folic acid tablet = 200–400 *μ*g/day. Placebo tablet	FA (100) = 21 FA (200) = 28 FA (400) = 26 *P* = 19	Double blind, identical tablets. Duration: 10 wks	Plasma homocysteine (*μ*mol/L) (automated fluorescence-polarization method).	

Lamers et al., 2004 [30]	Germany	Childbearing age women (144)	Folic acid capsule = 400 *μ*g/day. [6S]-5-MTHF capsule = 416 *μ*g/day. [6S]-5-MTHF capsule = 208 *μ*g/day. Placebo capsule	FA = 34 Low MTHF = 32 High MTHF = 35 *P* = 34	Double blind, hard gelatine capsules Duration: 24 wks	Plasma homocysteine (*μ*mol/L) (immunoassay on AxSYM analyser).	FA: ^a^ <0.01 Low MTHF: ^a^ <0.01 High MTHF: ^a^ <0.01

Lamers et al., 2006* [18]	Germany	Childbearing age women (144)	Folic acid capsule = 400 *μ*g/day. [6S]-5-MTHF capsule = 416 *μ*g/day. [6S]-5-MTHF capsule = 208 *μ*g/day. Placebo capsule	FA = 34 Low MTHF = 33 High MTHF = 35 *P* = 34	Double blind, hard gelatine capsules. Duration: 24 wks.	Red blood cell folate (nmol/L) ({(whole blood folate × 100) – (plasma folate × (100 – hematocrit))}). Plasma folate (nmol/L) (microbiological assay)	

Fohr et al., 2002 [31]	Germany	Childbearing age women (163)	Folic acid capsule = 400 *μ*g/day. MTHF (racemic mixture) capsule = 480 *μ*g/day. Placebo capsule	FA = 51 MTHF = 52 *P* = 57	Double blind. Duration: 8 wks.	RBC folate (nmol/L) (immunoassay kit for IMx analyser). Plasma folate (nmol/L) (immunoassay kit for IMx analyser). Plasma homocysteine (*μ*mol/L) (reversed-phase HPLC).	

Green et al., 2005 [32]	New Zealand	Childbearing age women (73)	75 g powdered milk daily. Fortified milk = 375 *μ*g folic acid/day. Control milk	FA = 36 *P* = 37	Double blind. Duration: 12 wks. Dietary assessment	RBC folate (nmol/L) (from whole blood folate by subtracting plasma folate and correcting for hematocrit). Plasma folate (nmol/L) (microtiter technique, chloramphenicol resistant, L. Casei). Plasma homocysteine (*μ*mol/L) (Imx analyzer).	

Ellison et al., 2004 [33]	UK	Pregnant women (30)	Folic acid capsule = 400 *μ*g/day. Control group: no treatment.	FA = 15 *P* = 15	Duration: 24 wks.	Plama folate (ng/mL). Plasma homocysteine (*μ*mol/L) (enzyme immunoassay).	<0.05

Lira et al., 1989 [34]	Spain	Pregnant women (153)	Multivitamin capsules. Supplement = 350 *μ*g folic acid, 105 mg ferrous sulphate, 500 mg ascorbic acid, daily. Placebo = 105 mg ferrous sulphate, 500 mg ascorbic acid, daily.	FA = 75 *P* = 78	Range: 0–350 *μ*g/day. Duration: 25 wks.	RBC folate (*μ*g/L) (L. Casei microbiological assay). Serum folate (*μ*g/L) (L. Casei microbiological assay).	<0.001 <0.001

Nogueira et al., 2003 [35]	Brazil	Pregnant women (114)	Mineral tablets. Supplement = 250 *μ*g folic acid, 120 mg iron sulphate/day. Placebo: 120 iron sulphate/day.	FA = 15 *P* = 16	Duration: 22 wks.	Plasma folate (mg/mL) (Radioimmunoassay-Iodo125).	

Houghton et al., 2006 [36]	Canada	Lactating women (69)	Folic acid capsule = 400 *μ*g/day. [6S]-5-MTHF capsule = 416 *μ*g/day. Placebo capsule	FA = 21 MTHF = 21 *P* = 22	Double blind. Duration: 16 wks. Dietary assessment	RBC (nmol/L) (from the whole-blood folate by subtracting plasma folate and correcting for hematocrit). Plasma folate (nmol/L) (L. rhamnosus microbiological assay). Plasma homocysteine (*μ*mol/L) (HPLC)	FA: <0.002 MTHF: <0.0001 MTHF: <0.002

Mackey and Picciano, 1999 [20]	USA	Lactating women (42)	Folic acid capsules = 1 mg/day. Placebo tablet	FA = 21 *P* = 21	Double blind, folic acid tablets indistinguishable from placebo tablets. Duration: 12 wks. Dietary assessment	RBC folate (nmol/L) (L. Casei microbiological assay). Plasma folate (nmol/L) (L. Casei microbiological assay). Plasma homocysteine (*μ*mol/L) (HPLC).	<0.05

*The Authors kindly requested us to cite both the publication in AJCN [18] and Lamers' Ph.D. thesis for the raw data [37].

RCB: red blood cell.

^a^Significant differences between the baseline and the end of the treatment.

FA: folic acid.

MTHF: methyltetrahydrofolate.

**Table 3:** Effects of total folate supply (i.e., folic acid* plus* dietary folate)^1^ on folate in plasma/serum and in red blood cells, as well as on plasma homocysteine levels in women, stratified by population group, duration of supplementation, and dose of folic acid supplementation.

Stratum for analysis	Folate in plasma/serum			Folate in red blood cells			Total plasma homocysteine		
	No. of studies (*n* participants)	Regression coefficient [95% CI]	Heterogeneity *I* ^2^ (%)	No. of studies (*n* participants)	Regression coefficient [95% CI]	Heterogeneity *I* ^2^ (%)	No. of studies (*n* participants)	Regression coefficient [95% CI]	Heterogeneity *I* ^2^ (%)
All studies	9 (632)	0.56*** [0.40, 0.72]	92	10 (724)	0.30*** [0.22, 0.38]	82	9 (585)	−0.10** [−0.17, −0.04]	72
Population group									
Women in childbearing age	4 (343)	0.65*** [0.39, 0.92]	96	7 (486)	0.33*** [0.23, 0.44]	86	6 (470)	−0.12*** [−0.15, −0.08]	0
Pregnant women	3 (204)	0.52*** [0.30, 0.75]	69	1 (153)	0.26*** [0.15, 0.37]	n.a	1 (30)	−0.42*** [−0.58, −0.25]	n.a
Lactating women	2 (85)	0.36 [0.08, 0.79]	90	2 (85)	0.19*** [0.09, 0.28]	0	2 (85)	0.03 [−0.04, 0.11]	0
Duration of supplementation									
4–12 weeks	4 (317)	0.51*** [0.22, 0.80]	96	6 (415)	0.27*** [0.17, 0.37]	83	6 (444)	−0.10*** [−0.14, −0.05]	31
13–20 weeks	1 (43)	0.58*** [0.38, 0.77]	n.a	1 (43)	0.26** [0.08, 0.44]	n.a	1 (43)	0.03 [−0.06, 0.12]	n.a
≥21 weeks	4 (272)	0.60*** [0.38, 0.82]	84	3 (266)	0.39*** [0.23, 0.55]	76	2 (98)	−0.26 [−0.55, 0.04]	90
Dose									
≤250 *μ*g FA/day	2 (120)	0.61* [0.05, 1.17]	82	2 (146)	0.28 [−0.06, 0.62]	70	2 (146)	−0.13 [−0.28, 0.02]	68
251–500 *μ*gFA/day	7 (569)	0.59*** [0.41, 0.76]	93	9 (682)	0.32*** [0.23, 0.40]	82	8 (543)	−0.12*** [−0.18, −0.06]	69
>500 *μ*g FA/day	1 (42)	0.13 [−0.07, 0.33]	n.a	1 (42)	0.16** [0.04, 0.27]	n.a	1 (42)	0.05 [−0.08, 0.18]	n.a

^1^ The amounts of folic acid from supplements/fortified foods were transformed into amounts of folate by multiplying × 1.7 (IOM, 2000; [6]). When not provided in the RCT, the dietary folate intake used corresponded to the mean value of 247 *μ*g/day.

n.a: not applicable.

Test for overall effect (*P*): *<0.05, **<0.01, ***<0.001.

**Table 4:** Forest plot of the effects of total folate supply (i.e., supplement *p*l*us* dietary folate), with the supplement provided in form of folic acid and [6S]-5-methyltetrahydrofolate [5-MTHF] on serum or plasma folate in childbearing age, pregnant and lactating women.

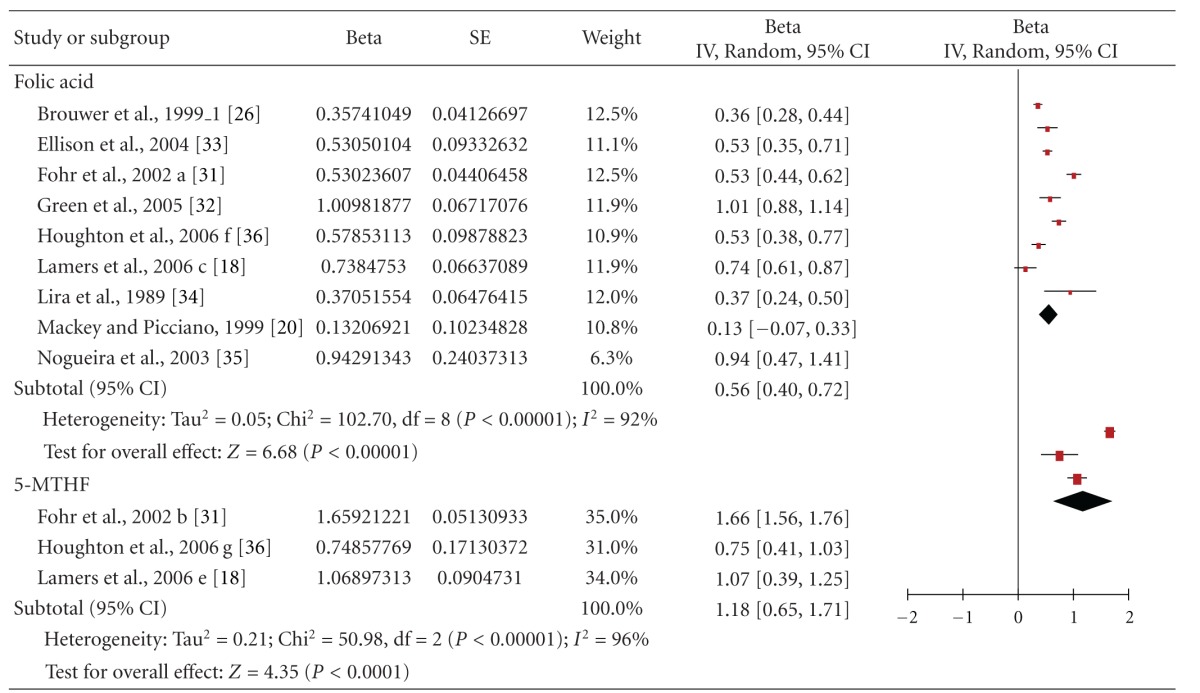

*The Authors kindly requested us to cite both the publication in AJCN [18] and Lamers' Ph.D. thesis for the raw data [37].

RCB: red blood cell.

^a^Significant differences between the baseline and the end of the treatment.

FA: folic acid.

MTHF: methyltetrahydrofolate.

**Table 5:** Forest plot of the effects of total folate supply (i.e., supplement plus dietary folate), with the supplement in form of folic acid and [6S]-5-methyltetrahydrofolate (5-MTHF) on red blood cell (RBC) folate in childbearing age, pregnant and lactating women.

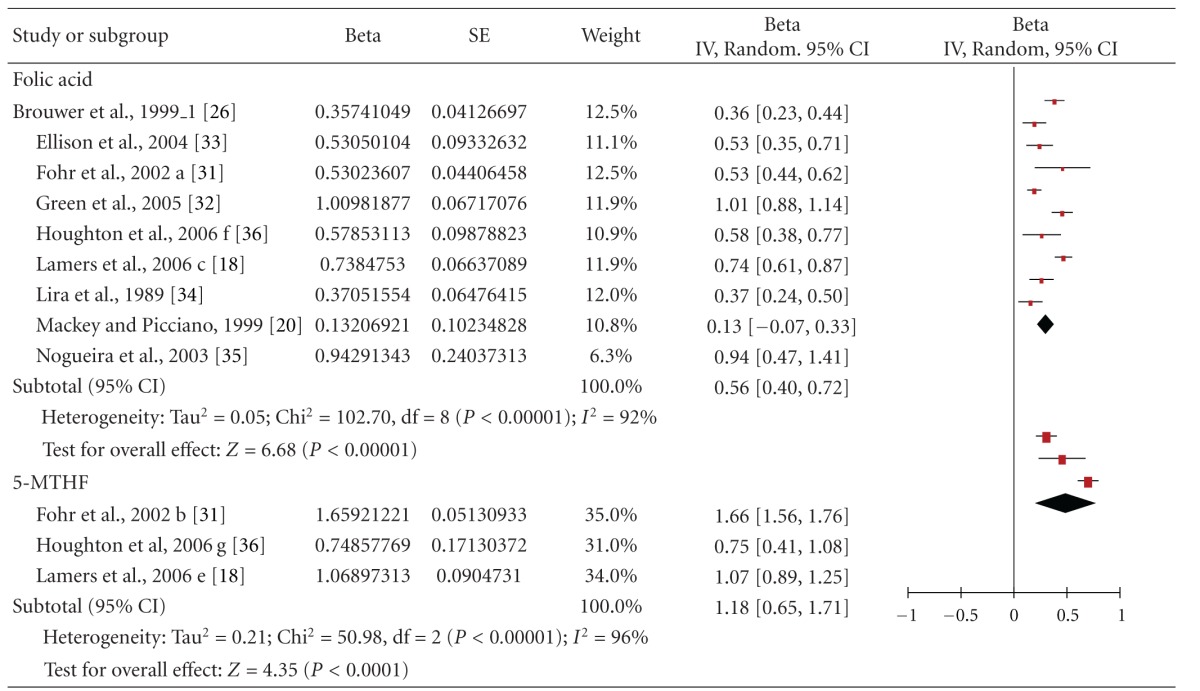

*The Authors kindly requested us to cite both the publication in AJCN [18] and Lamers' Ph.D. thesis for the raw data [37].

RCB: red blood cell.

^a^Significant differences between the baseline and the end of the treatment.

FA: folic acid.

MTHF: methyltetrahydrofolate.

**Table 6:** Forest plot of the effects of total folate supply (i.e., supplement plus dietary folate), with the supplement provided in form of folic acid and [6S]-5-methyltetrahydrofolate (5-MTHF) on plasma homocysteine (tHcy) folate in childbearing age, pregnant and lactating women.